# A Case Report and an Overview of the Differential Diagnosis of Pigmented Lesions of the Conjunctiva: Just a Freckle or Something to Heckle?

**DOI:** 10.7759/cureus.66559

**Published:** 2024-08-10

**Authors:** Margarida Ribeiro, Pedro Marques-Couto, Ana Gama-Castro, Teresa Dinah-Bragança, João Barbosa-Breda

**Affiliations:** 1 Department of Ophthalmology, Unidade Local de Saúde de São João, Porto, PRT; 2 Department of Biomedicine, Unit of Pharmacology and Therapeutics, Faculty of Medicine of the University of Porto, Porto, PRT; 3 Cardiovascular Research and Development Centre – UnIC@RISE, Department of Surgery and Physiology, Faculty of Medicine of the University of Porto, Porto, PRT; 4 Research Group Ophthalmology, Department of Neurosciences, KU Leuven, Leuven, BEL

**Keywords:** ophthalmology, eye, primary acquired melanosis, complexion-associated melanosis, conjunctival melanoma, nevus

## Abstract

A 42-year-old darkly pigmented woman presented at our emergency department with complaints of pruritus, foreign body sensation, and blurry vision. Besides findings compatible with mild dry eye disease, anterior segment examination revealed dark pigmented, ill-defined areas of the conjunctiva surrounding the temporal limbus and temporal bulbar conjunctiva in the left eye and involving the superior and inferior tarsal conjunctiva in both eyes. The patient reported no recent changes in these lesions. A diagnosis of complexion-associated melanosis in the palpebral conjunctiva of both eyes and primary acquired melanosis in the bulbar conjunctiva of the left eye was assumed, and the patient was placed under regular follow-up. Preservative-free tears were also recommended for her dry eye condition.

Conjunctival pigmented lesions are a common finding during ophthalmologic evaluations. They pose a significant diagnostic challenge due to the wide array of differential diagnoses and the concern of misdiagnosing melanoma. A comprehensive clinical history and multidisciplinary evaluation are crucial in managing these cases.

## Introduction

The conjunctiva is a thin mucous membrane that covers the anterior part of the eye. It can be divided into four zones, namely, the tarsal, forniceal (these two correspond to the palpebral conjunctiva), bulbar, and caruncular conjunctiva [1,2]. As in the skin, it contains resident melanocytes within the basal layer of the epithelium [3]. Various non-pigmented and pigmented lesions, ranging from benign to malignant, can affect the conjunctiva. The most common type of conjunctival neoplasms is melanocytic, although this might be explained by referral bias, due to their higher visibility during the physical exam [4]. Pigmented lesions that arise from the conjunctiva include nevus, complexion-associated melanosis (CAM), primary acquired melanosis (PAM), and melanoma [5].

This paper intends to report a case of a patient with conjunctival CAM and to provide an overview of the differential diagnosis and management of pigmented lesions of the conjunctiva.

## Case presentation

A 42-year-old darkly pigmented woman presented at the ophthalmology emergency department with complaints of pruritus, foreign body sensation, and blurry vision for two weeks. The patient reported regular use of mascara.

**Figure 1:**
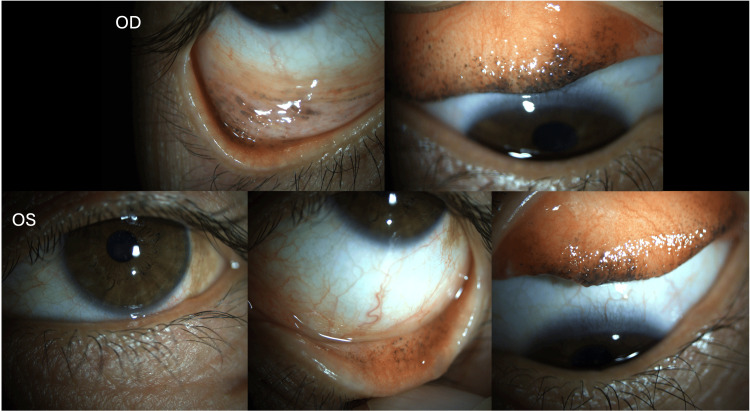
Photographs of the pigmented lesions of the conjunctiva in the right and left eyes after lid eversion. Admission photographs demonstrating several bilateral and symmetric dark pigmented, ill-defined areas involving the superior and inferior tarsal conjunctiva in both eyes, which can be compatible with complexion-associated melanosis. Additionally, one golden yellow to brown dusted pigment lesion with two clock hours of extension in the bulbar conjunctiva surrounding the temporal limbus in the left eye is suggestive of primary acquired melanosis. OD: right eye; OS: left eye.

Upon ophthalmologic examination, her best-corrected visual acuity was 10/10 in the right eye (OD) and 9/10 in the left eye (OS). Anterior segment examination revealed several dark pigmented, ill-defined areas involving the superior and inferior tarsal conjunctiva in both eyes (Figure 1). Additionally, one more pigmented lesion with two clock hours of extension was noticed in the bulbar conjunctiva surrounding the temporal limbus in the OS. There was also a mild punctate keratitis bilaterally. The remaining anterior segment examination was unremarkable. Evaluation of the posterior segment showed no abnormalities and intraocular pressure was within normal limits.

Upon further questioning, the patient denied any recent changes in the pigmented lesions and reported no familiar history of skin or ocular cancer.

After a multidisciplinary discussion involving the ophthalmic oncology section of our department, a diagnosis of CAM was assumed. Since there was no recent evolution of the lesions, confirmatory histologic analysis was deferred and the patient continued follow-up with the ophthalmology clinic for surveillance of the lesion’s behavior, taking special care to the unilateral lesion observed in the bulbar conjunctiva in the OS, since it was not possible to exclude PAM. Mascara eviction was recommended and preservative-free tears were provided for her dry eye condition.

## Discussion

In general, lesions of the bulbar conjunctiva are more common and apparent than those in the fornix or in the tarsal conjunctiva, which can be missed by the patient, the primary care (PC) physician, and the ophthalmologist if a meticulous examination of the eyelids after eversion is not performed.

Melanocytic lesions that arise from the conjunctiva include nevus, CAM, PAM, and melanoma. Although nevus carries a small risk of malignant transformation, this risk is negligible in CAM. On the other hand, PAM with atypia has a risk of progression to malignant melanoma of up to 50% [6]. Questions regarding the age when the patient first noted the lesion, history of sun exposure, family or personal history of skin cancer, or if the lesion has evolved in color, thickness, or size are fundamental during the anamnesis. Incidental pigment deposition due to the use of different products, such as mascara, must be excluded since it is an important differential diagnosis [7]. Clinical features of each entity should be acknowledged by any comprehensive ophthalmologist and ideally by the PC physician, who should be able to identify high-risk features and immediately refer patients to the ophthalmology department.

Nevi are the most common conjunctival melanocytic lesions (more than 50%) [4,6,8]. They usually appear in childhood and are typically located in the interpalpebral conjunctiva (part of the bulbar conjunctiva), near the limbus, and they remain relatively stationary throughout life. This lesion is more frequent in the bulbar conjunctiva, although it can appear in other locations. They are well-circumscribed, slightly raised, and have a cystic appearance. In most cases, nevi can just be followed annually with photo documentation. They should not require removal unless any changes in color or size are noticed [9]. However, those do not necessarily indicate the development of melanoma, as they may occur with hormonal changes such as in puberty and pregnancy [1].

CAM or racial melanosis is a benign condition observed in darkly pigmented individuals [6]. According to a recent population-based study in Minnesota, USA, the adjusted incidence of CAM was 10.8 per 1,000,000 person-years [10]. It is typically a flat, noncystic lesion with ill-defined margins and a “cobblestone” appearance observed around the limbus [6]. In contrast with nevi, CAM can grow extensively with aging, often extending 360 degrees around the limbus [3]. This condition is typically bilateral and symmetrical, distinguishing it from nevi and PAM [11]. Although rare, it might involve the palpebral conjunctiva [3]. In this case, we observed several symmetrical and bilateral lesions involving the superior and inferior palpebral conjunctiva after performing lid eversion. Although CAM is not a risk factor for melanoma, darkly pigmented individuals can also develop PAM and conjunctival melanomas, and therefore regular observation is recommended in these patients [3,6].

This patient also had a unilateral pigmented lesion in the bulbar temporal conjunctiva in the OS, for which regular ophthalmologic follow-up was decided to monitor its behavior since it was not possible to exclude PAM. Therefore, even the most seemingly benign lesions require careful evaluation [6]. PAM is most likely observed in fair-skinned individuals who are middle-aged or older, but it can develop at any age and any race [3,6]. According to a series of 5002 conjunctival tumors referred to a tertiary ocular oncology center, the prevalence of PAM was found to be 12% [4]. A unilateral, heterogenous, patchy, golden yellow to brown mottled or dusted pigment is observed. Its appearance regarding size and degree of pigmentation can change over time. It is most often observed in the bulbar conjunctiva (around 90%), but can also occur in the palpebral conjunctiva, corneal epithelium, fornix, and caruncula [3,6]. This condition is clinically different from nevi, which are much more well-circumscribed, non-progressive, and present in younger ages [6,12]. Small lesions (less than one to two clock hours) can be monitored annually, while larger lesions should be promptly excised followed by histologic examination [3,13]. Only with pathology findings can PAM be identified as PAM with or without atypia. The former has no potential risk of malignant transformation, while the latter may have a risk of up to 50% [6]. Given that the lesion observed in this case is small and not very dense, regular surveillance is appropriate. The risk of melanoma progression depends mainly on the size of the lesion. Serial observation is required approximately every six months with photographic documentation [6]. Larger lesions require excision and histologic analysis. Likewise, if there is a history of conjunctival melanoma or nodular areas and/or increased vascularity are present within a pigmented conjunctival lesion presumed to be PAM, a more aggressive approach is warranted, as these features increase the likelihood that PAM may be a precursor/early melanoma [3].

In contrast to choroidal melanoma (the most common form of ocular melanoma), conjunctival melanoma is a very rare but also extremely serious and life-threatening condition with an estimated incidence of 0.5 per one million people per year [14], and a mortality rate of 8% at 10 years of follow-up [15]. Nearly 20% (19%) of patients with conjunctival melanoma develop metastatic disease at 10 years [15]. Metastases to ipsilateral facial lymph nodes, brain, lung, and liver are the most common sites [16]. It is suggested that conjunctival melanoma is associated with UV exposure and it is more common in fair-skinned individuals. Patients with conjunctival melanoma are typically middle-aged adults (5th to 6th decade of life) [3] and present with a nodular mass arising either de novo (carrying the worst prognosis), from a nevus or PAM with atypia (most common, observed in 75%). General features often seen at the slit lamp are elevation of the lesion, immobility, and vascularity (feeder vessels). Pigmentation is present in the vast majority of patients, but it can be also non-pigmented [6]. This lesion most frequently involves the bulbar and limbal conjunctiva, but can also appear in the palpebral conjunctiva, fornix, and caruncle, and these three locations are associated with worse prognosis [3,6]. The gold standard of treatment relies on complete surgical excision with wide clear margins (3-4 mm) followed by cryotherapy and/or topical chemotherapy. Systemic therapy, enucleation, exenteration, and radiation are used in advanced and/or cases deemed inappropriate for surgical intervention [3]. This diagnosis implies prompt staging and metastatic workup in conjunction with the oncologist [14].

## Conclusions

Melanocytic lesions of the conjunctiva can be challenging. Several possible diagnoses should be considered, varying from innocuous conditions such as incidental pigment deposition and CAM to malignant and life-threatening conditions such as melanoma. Although the clinical features may overlap, the prognosis and management strategies are very distinctive. Therefore, we recommend that any conjunctival pigmented lesion noticed by the primary care physician should be referred to the ophthalmologist. Additionally, although these lesions appear most commonly in the bulbar conjunctiva, they may be hidden in the palpebral conjunctiva. Therefore, a simple gesture such as lid eversion should always be part of the basic initial evaluation of an ophthalmologist.

## References

[1] Bresler SC, Simon C, Shields CL, McHugh JB, Stagner AM, Patel RM (2022). Conjunctival melanocytic lesions. Arch Pathol Lab Med.

[2] Kheir WJ, Tetzlaff MT, Pfeiffer ML, Mulay K, Ozgur O, Morrell G, Esmaeli B (2016). Epithelial, non-melanocytic and melanocytic proliferations of the ocular surface. Semin Diagn Pathol.

[3] Whittington CP, Bresler SC, Simon C, Shields CL, Patel RM (2024). Melanocytic lesions of the conjunctiva: an up-to-date review. Diagn Histopathol.

[4] Shields CL, Alset AE, Boal NS (2017). Conjunctival tumors in 5002 cases. Comparative analysis of benign versus malignant counterparts. The 2016 James D. Allen lecture. Am J Ophthalmol.

[5] Sayyad Sayyad, F.F.E. and Karp, C.L. (2006 (2013). Ophthalmic pearls. Conjunctival pigmented lesions: diagnosis and management. https://www.aao.org/eyenet/article/conjunctival-pigmented-lesions-diagnosis-managemen.

[6] Oellers P, Karp CL (2012). Management of pigmented conjunctival lesions. Ocul Surf.

[7] Alevi D, Donnenfeld E, Perry H (2016). Mascara-induced conjunctival pigmentation. JAMA Ophthalmol.

[8] Shields CL, Demirci H, Karatza E, Shields JA (2004). Clinical survey of 1643 melanocytic and nonmelanocytic conjunctival tumors. Ophthalmology.

[9] Levecq L, De Potter P, Jamart J (2010). Conjunctival nevi clinical features and therapeutic outcomes. Ophthalmology.

[10] Dalvin LA, Salomão DR, Patel SV (2018). Population-based incidence of conjunctival tumours in Olmsted County, Minnesota. Br J Ophthalmol.

[11] Shields CL, Shields JA (2004). Tumors of the conjunctiva and cornea. Surv Ophthalmol.

[12] Shields CL, Fasiuddin AF, Mashayekhi A, Shields JA (2004). Conjunctival nevi: clinical features and natural course in 410 consecutive patients. Arch Ophthalmol.

[13] Shields JA, Shields CL, Mashayekhi A (2007). Primary acquired melanosis of the conjunctiva: experience with 311 eyes. Trans Am Ophthalmol Soc.

[14] Shields CL, Silva AM, Laiton A (2024). Conjunctival melanoma: insights into classification, outcomes, and biomarkers. Clin Dermatol.

[15] Shields CL, Yaghy A, Dalvin LA (2020). Conjunctival melanoma: outcomes based on the American Joint Committee on Cancer Clinical Classification (8th Edition) of 425 patients at a single ocular oncology center. Asia Pac J Ophthalmol (Phila).

[16] Shields CL, Shields JA (2019). Tumors of the conjunctiva and cornea. Indian J Ophthalmol.

